# Estimation of Temporal Gait Parameters Using a Human Body Electrostatic Sensing-Based Method

**DOI:** 10.3390/s18061737

**Published:** 2018-05-28

**Authors:** Mengxuan Li, Pengfei Li, Shanshan Tian, Kai Tang, Xi Chen

**Affiliations:** State Key Laboratory of Mechatronics Engineering and Control, Beijing Institute of Technology, Beijing 100081, China; formlmx@126.com (M.L.); pfli@bit.edu.cn (P.L.); shanshanbit@126.com (S.T.); tangkai0205@163.com (K.T.)

**Keywords:** electrostatic field sensing, gait measurement, temporal parameters

## Abstract

Accurate estimation of gait parameters is essential for obtaining quantitative information on motor deficits in Parkinson’s disease and other neurodegenerative diseases, which helps determine disease progression and therapeutic interventions. Due to the demand for high accuracy, unobtrusive measurement methods such as optical motion capture systems, foot pressure plates, and other systems have been commonly used in clinical environments. However, the high cost of existing lab-based methods greatly hinders their wider usage, especially in developing countries. In this study, we present a low-cost, noncontact, and an accurate temporal gait parameters estimation method by sensing and analyzing the electrostatic field generated from human foot stepping. The proposed method achieved an average 97% accuracy on gait phase detection and was further validated by comparison to the foot pressure system in 10 healthy subjects. Two results were compared using the Pearson coefficient *r* and obtained an excellent consistency (*r* = 0.99, *p* < 0.05). The repeatability of the purposed method was calculated between days by intraclass correlation coefficients (ICC), and showed good test-retest reliability (ICC = 0.87, *p* < 0.01). The proposed method could be an affordable and accurate tool to measure temporal gait parameters in hospital laboratories and in patients’ home environments.

## 1. Introduction

Human gait is a coordinated and rhythmic periodic motion produced by an integrated control of nerve and muscle systems, which is mainly dictated by body features and health conditions. The gait cycle can be defined as the time interval between two initial contacts of the same foot. The initial contact (IC) and separate events (SE) segment the gait cycle into gait phases and provide temporal gait parameters. Temporal gait parameters are essential in many gait analysis applications [1], such as (1) the evaluation of rehabilitation status in patients [2,3]; (2) the recognition of daily life activities [4]; and (3) distinguishing between a normal and pathological gait [5].

The technological devices used to acquire human gait can be classified into two categories: those based on non-wearable sensors and those based on wearable sensors [6]. Non-wearable sensor systems require lab-based facilities where the sensors are located and capture data on the gait while the subject walks on a certain space. Typical representatives of non-wearable sensors systems are the foot pressure plate [7] and the optical motion capture system. They are often used as the gold standard for measuring gait parameters. However, these measurement methods still have a certain degree of limitation when it comes to commercial measurement products, which are generally expensive. For example, the market price of the pressure plate is about $2000. Non-commercial laboratory self-made measurement equipment has relatively complex components such as the walkway in Reference [8]. More than 18,432 sensors arranged under a rubber pad were used for parameter acquisition. They are bulky and only suited to capture human gait in a confined space. The optical motion capture system has the disadvantage of light interference and uses complicated processing algorithms. Furthermore, laboratory settings are unable to represent the daily environment. For this reason, the subject who is performing the test will change their inherent gait rhythm due to the fact that they may feel conditioned by the specialist and the experiment system. The above defects greatly restrict the extensive usage of these methods, especially in developing countries.

In contrast, wearable sensor systems make it possible to analyze data outside the laboratory and capture gait parameters during a person’s daily activities. The wearable sensor systems use sensors attached to several parts of the body such as the ankles, knees, or waist. Different types of sensors are used to capture gait signals and extract gait parameters. These include accelerometers [9], gyroscopic sensors [10], electromyography [11], and more. These gait analysis platforms are widely considered to be viable alternatives and are made possible by the recent progress of integrated circuits and sensing technology. The prospect of these sensors is to simplify gait analysis, making it relatively cheaper and no longer constricted to use in a laboratory. Accelerometers and other inertial sensors have been widely used in gait events estimation [12], gait action recognition [13], and gait analysis because they are miniaturized, low powered, durable, inexpensive, highly mobile, and readily available [14,15,16,17,18]. Clearly, low-cost wearable accelerometer sensors can be easily integrated into the acts of daily living and are beneficial for pre-diagnostic and rehabilitation monitoring [19]. With the online gait event detection algorithm embedded into this device, it simplified the analysis and acquisition of the gait parameters. This analysis may include operations such as the segmentation of gait signals into gait cycles, the estimation of temporal gait parameters [20], and the calculation of gait asymmetry, gait variability, and gait stability [21].

However, accelerometers are susceptible to researchers’ placement skill and minor variations in the attachment site. The same sensor placed in five anatomical foot locations could obtain different evaluation accuracies of gait parameters [22]. Moreover, installation error will also lead to an inaccuracy of the gait parameter measurement. Unlike accelerometers, careful placement of the sensor on the body segments is not mandatory when using gyroscopic sensors [10]. When using the sensor output of different positions of the tester especially on the shank and thighs, more than 90% of the temporal gait parameters’ measurement accuracy can be obtained [23]. However, these wearable sensors share a defect, in that they all require devices to be placed on the subject’s body, which may be uncomfortable or obtrusive. Moreover, wireless systems usually store data on SD cards or transmit data through Bluetooth or Zigbee devices to personal computers, which demands a high energy consumption. The most commonly used energy sources are lithium batteries and, if gait is to be monitored over a long period of time, the capacity of the batteries may be a problem.

Due to this background, there is a need for affordable non-wearable and long-time monitoring technologies for gait analysis, which may be capable of continuously monitoring gait parameters during daily activities and reducing the stress and anxiety in testers subjected to gait studies. Such systems can accurately acquire a certain number of gait parameters and identify changes in gait patterns. Due to this, specialists can use the information to predict adverse events and diagnose early symptoms of some specific diseases, which encourages timely medical interventions. Accordingly, these technologies can complement traditional gait analysis systems.

Actually, electrostatic field sensing (EFS) technology may be an effective solution to this problem. The use of a non-contact EFS method for gait measurement has become a hot topic in recent years. In Reference [24], Koichi Kurita measured the electrostatic signals when walking with the electrostatic induction method and a correlation analysis of the gait signals was implemented by combining with the frequency domain information of the signal. We established the human body equivalent capacitance model through theoretical derivation, verified the correctness of the model through simulation, and measured the gait signal [25]. Previous studies on the human body electrostatic sensing method have proven that the gait signal can be obtained by the EFS method. However, no studies have explored how to extract the temporal parameters from the gait signal nor conducted an investigation on the validity and repeatability of this method.

In this study, an algorithm for extracting temporal gait parameters in the EFS gait signal was suggested. The effectiveness of the method was verified by comparison with the results of the foot pressure system. The repeatability of the EFS method was calculated between days by intraclass correlation coefficients (ICC), and showed a good test-retest reliability. The rest of the paper is organized as follows. In Section 2, we present the principle of the EFS method, the details of the system setup, the algorithm, and the data collection procedures. In Section 3, we briefly show the results and the error comparison of two measurement methods. In Section 4, we discuss the results of the validation and repeatability study. The paper is concluded in Section 5, with final remarks and future research directions.

## 2. Method

### 2.1. Principle of Electrostatic Field Sensing

**Figure 1:**
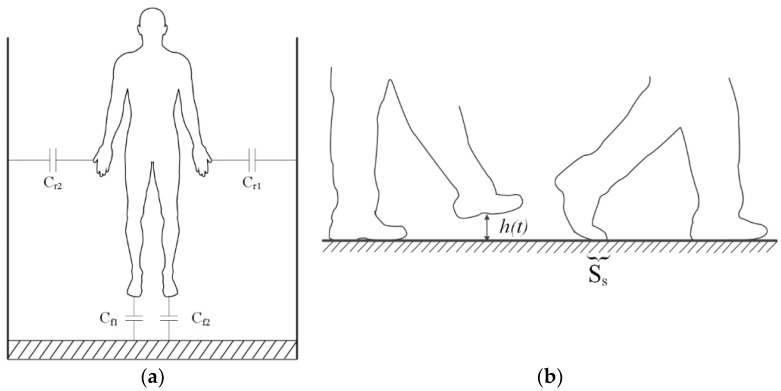
Schematic diagram of the human body movement capacitance model. (**a**) Schematic diagram of the human body equivalent capacitance; (**b**) Foot movement capacitance diagram.

The human body becomes charged with static electricity due to the creation of friction between the body and clothing [26,27,28]. Furthermore, friction, contact, and separation between the human foot and ground during walking also charges the human body [29,30,31]. The phenomenon of the human body becoming electrically charged leads to a corresponding change in the electric field around the human body with the foot movement during walking [32,33]. Based on this, we established an equivalent capacitance model of the human body and the surrounding environment [25]. When the human body is in a certain space, due to a certain amount of charge on the body, the human body and the surrounding environment produces an equivalent capacitance—including the left and right foot capacitance *C_f_*_1_ and *C_f_*_2_—to the ground when standing still and *C_ri_* (*i* = 1, 2, ...) between other parts of the human body and the surrounding environment, which together constitute the total capacitance of the human body (Equation (1)), where *C_f_ = C_f_*_1_ *+ C_f_*_2_ *= 2ε_s_S_s_*/*d_e_*. Here, *ε_s_*, *S_s_*, and *d_e_* are the dielectric constant of the shoe sole, the equivalent area of the floor capacitance formed by the shoe sole and ground, and the thickness of the shoe sole. The human body equivalent capacitance model is shown in Figure 1a.

(1) $$C_{h}=C_{f}+\sum_{i=1}^{\infty }C_{ri}$$

Suppose the human body charge is *Q_B_* and the two feet alternately leave the ground during walking. Then the capacitance between the foot and the ground is equivalent to *C_f_* in series with a capacitance that changes based on the height of the foot from the ground. Although *C_f_* cannot be measured directly, the law of human foot movement can be obtained through the analysis of changes in the induced current because the changes in capacitance caused by human body movement are directly related to the electrostatic induction current signal. Figure 1b shows two typical foot states in the process of the human body walking. In the figure, the height function of the foot from the ground in walking is defined as *h*(*t*), the equivalent area function of the plate capacitor formed by the foot and the ground is *S_s_*, and the air dielectric constant is *ε_a_*. The induced potential of the human body can be expressed by the equation below.

(2) $$U_{B}=Q_{B}\cdot \frac{\varepsilon_{a}S_{s}+h(t)C_{f}}{C_{f}\varepsilon_{a}S_{s}}$$

Assume the coupling capacitance between the human body and the induction electrode is *C*. Then, the charge *Q* induced by the induction electrode can be defined as below.

(3) $$Q=C(U_{B}-V)$$

where *V* is the induced potential on the induction electrode. Then, the induced current *I* flowing through the induction electrode can be deduced from the above equation.

(4) $$I=\frac{dQ}{dt}\;=C\frac{dU_{B}}{dt}\propto \frac{1}{S_{s}}\frac{d}{dt}(h(t))-\frac{h(t)}{{S_{s}}^{2}}\frac{dS_{s}}{dt}$$

Here, we assume that the human body is a good conductor. Equation (4) shows that when the tester walks in the vicinity of the induction electrode, the change in the induced current caused by human body movement can be measured under non-contact conditions. The temporal gait parameters can be obtained by analyzing the induced current waveform.

### 2.2. Instrumentation and Configurations

#### 2.2.1. Electrostatic Field Sensing Measurement Installation

**Figure 2:**
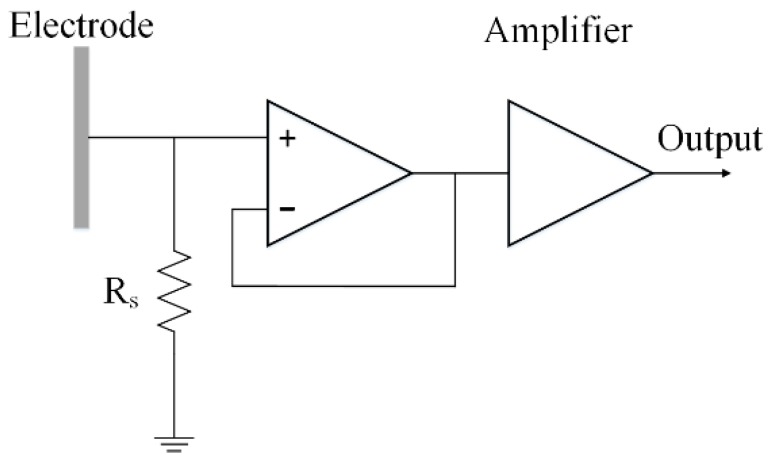
Schematic diagram of the I–V conversion part of the electrostatic measurement installation.

The induced current is generated on the induction electrode when the charged human body moves in the vicinity of the induction electrode of the measurement installation. The induced current is then converted to an observable voltage signal (conversion rate of 10 mV/pA), which is shown in Figure 2 through I–V conversion via the sampling resistor *R_s_* (*R_s_* is 10 GΩ). The voltage signal is then amplified by an operational amplifier (op-amp) for observation. Since the voltage signal obtained by this measurement method is easily disturbed by the power frequency voltage of the grid, we designed a low-pass filter with a cutoff frequency of 20 Hz before the analog-to-digital (A/D) conversion. Subsequently, the analog signal is converted into the digital signal through A/D for data acquisition and processing. The sampling rate adopted in this paper is 1 kHz. The digital signal obtained after A/D sampling is sent to a personal computer for data processing through the 2.4 GHz data transmission module.

EFS is a passive detection method. Its only response is to the changes of the surrounding electric field. The electric field disturbance caused by human foot movement is a low-frequency electric field with a typical frequency of 1~2 Hz. Based on this, we designed a low-pass filter with a cutoff frequency of 20 Hz to eliminate the influence of the high-frequency electric field on our measurement installation. When the measurement installation is carried out in a room with various kinds of measuring instruments and personal computers, the low-pass filter can effectively filter the interference of the high-frequency electric field produced by the electrical apparatus and the frequency of the power grid and can effectively obtain the low-frequency electric field signal produced by human motion. In order to avoid the influence of other individuals on the electric field tester, it is necessary to ensure that no other personnel walk in the effective measurement range of the measurement installation.

**Figure 3:**
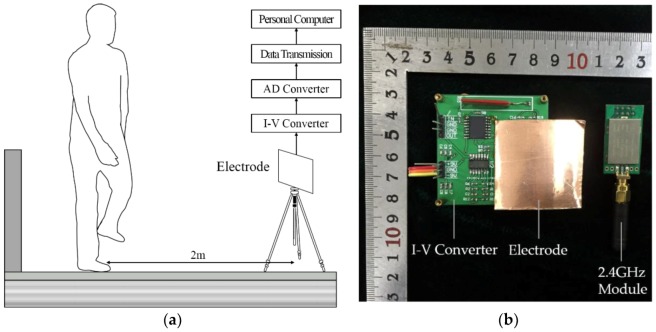
Illustration diagram and prototype of electrostatic measurement installation. (**a**) Illustration diagram of electrostatic measurement installation; (**b**) prototype of electrostatic measurement installation.

In the experiment, it is observed that the induced current flowing through the induction electrode is inversely proportional to the distance between the human body and the induction electrode. After repeated adjustment of the measurement installation sensitivity, the effective measurement distance of installation was adjusted to 3 m so that it can effectively measure the human body gait electrostatic signal without electric field interference by external equipment. In addition, the intensity of the induced current is related to the angle between the human body and the induction plate. The test results show that when the coronal plane of the human body is parallel to the electrode, the induced current is at a maximum level, as shown in Figure 3a. The induced current signal will be weakened when the angle between the human body and the induction plate changes, but the reduced amplitude change is not significant. A diagram illustrating the electrostatic measurement installation is shown in Figure 3a. The prototype of the electrostatic measurement installation is shown in Figure 3b.

#### 2.2.2. Foot Pressure Measurement System

In the design in Reference [34], we established a foot pressure system using two FSR406 pressure sensors of Interlink Electronics. This sensor features a low thickness (<0.05 inches), high dynamic response speed, insensitivity to temperature changes, resistance to high overload, and other advantageous characteristics.

Two pressure sensors with an effective area of 1.5 inches × 1.5 inches are fixed to the bottom of the insoles with medical tape and the insoles are cut from the transparent folder according to the foot bottom contour. One of the sensors is fixed in the front of the insole and roughly positioned under the toes while the other sensor is fixed in the rear part of the insole and roughly positioned under the heel. The two pressure sensors are connected in parallel and are actually used as a single pressure sensor. The system is powered by a 5 V battery. The two sensors are connected in series with the divider resistance and the changes in voltage values caused by pressure change are amplified by the operational amplifier before output. Experimental results show that the signal output is in the range of 0~2.5 V. The conditioning circuit together with the battery is fixed on the tester’s legs. The signal output after A/D conversion is sent to a personal computer for data processing with a 2.4 GHz data transmission module.

### 2.3. Algorithm Development

#### 2.3.1. Pressure-Based Foot Events Calculation Algorithm

**Figure 4:**
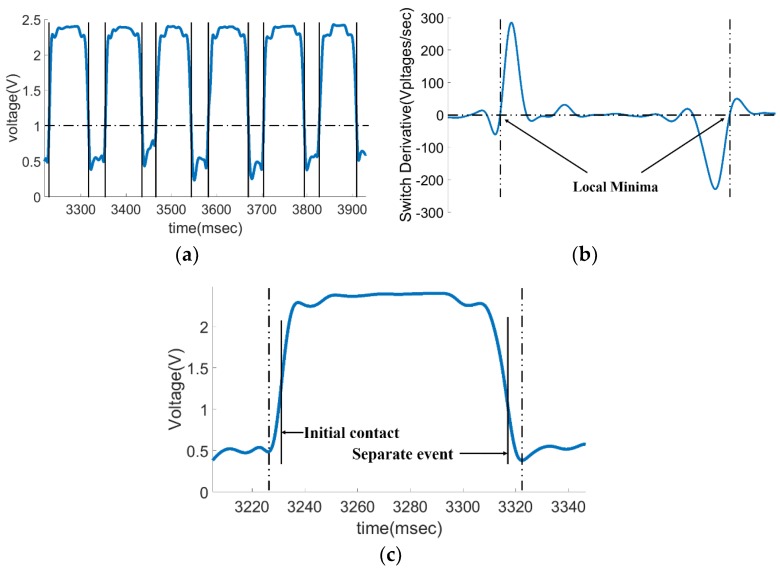
The process extracting the start and end moments of the support phase from the data measured by the foot pressure test system. (**a**) Pressure data time-domain waveform measured at normal speed by the foot pressure system as well as the rising edge and the falling edge of the signal from the threshold judgment; (**b**) signal local minimum point determined through the derivation of the signal in one cycle; (**c**) the initial contact and separate event of the gait cycle obtained by offsetting the minimum point.

The algorithm employed to determine the initial contact and separate events of the gait is divided into three steps. Since the pressure sensor output voltage is proportional to the foot pressure, we first determine the pressure time zone when the human foot is in the stance phase using the output voltage threshold. Afterward, we search for the minimum pressure signal near the threshold point, which is an important reference for determining the initial contact and separate events of the gait. Lastly, the time point determined in the previous step is compensated to determine the final contact and separation moments. The specific algorithm is described as follows. (1) Determine the time zone of the gait stance phase according to the rising and falling edges of the signal. The monotonically increasing signal above the fixed threshold can be determined as a rising edge and the monotonically reducing signal below the fixed threshold can be determined as a falling edge. In this paper, the threshold selected is 1 V, which is much smaller than the full load output of the pressure sensor when the foot fully contacts the ground and is much larger than the no load output; (2) Find out the minimum value of the signal in the rising and falling edges of the signal. With the human body walking movement, the output value of the pressure sensor under no load may drift. Therefore, we cannot use a fixed output value as the standard by which one can judge a moment. To find out the minimum value, we can derive the signal and find out the zero point from the peak point of the derivative, as shown in Figure 4b; (3) Lastly, the contact and separation moments are corrected by the local minimum point of the signal. The specific offset is 3% of the difference between the local maximum value and the local minimum value of the voltage signal in a gait cycle, and the local minimum point obtained in the previous step is shifted. The signal output, its derivative, and local minimum values, as well as the gait contact and separation moments determined by the offset during a test, are shown in Figure 4.

#### 2.3.2. Electrostatic Signal-Based Foot Events Calculation Algorithm

**Figure 5:**
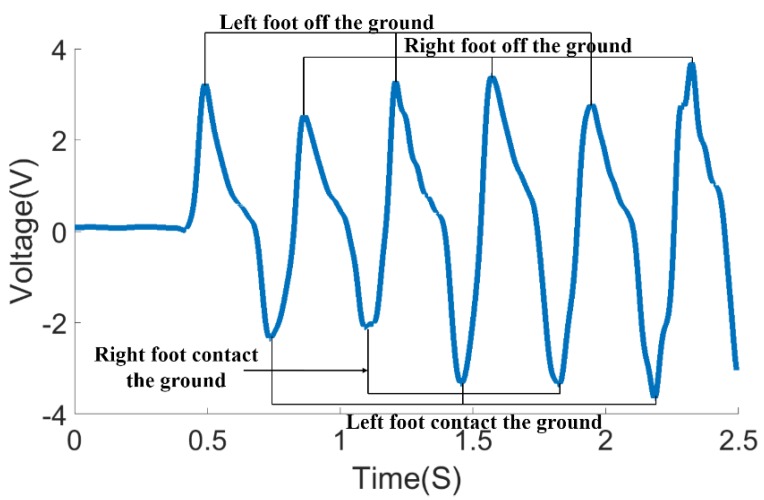
Time-domain waveform of electrostatic sensing signal.

In one test process, the time-domain waveform obtained by the electrostatic sensing system is shown in Figure 5.

Figure 5 shows the changes in body potential as the human body comes in contact with and is separated from the ground during stepping movements. The time-domain waveform includes the time parameters during the stepping movement. It can be determined using Equation (4) that the denominator of the left term is *S_s_* and the denominator of the right term is *S_s_*^2^. Therefore, the right-hand term affects slightly more than the induced current *I* and the left-hand term mainly affects the induced current *I*. For example, when the right foot toe is about to leave the ground, the equivalent area *S_s_* between the sole and the ground decreases. The distance *h*(*t*) between the right foot and the ground increases, so the induced current through the induction electrode increases when the right foot leaves the ground. The rate of change of *h*(*t*) is at the maximum level and the electrostatic signal reaches the local maximum level. Therefore, the results are consistent with the forecast from Equation (4). Subsequently, the induced current decreases as the lifting speed slows down. In the latter part of the swing phase, *h*(*t*) decreases rapidly and leads to the decrease of the induced current, which is consistent with the prediction made by the left side of Equation (4). The equivalent area *S_s_* of the heel contact area increases, which results in a rapid decrease in the induced current. Therefore, Equation (4) can effectively describe the variation trend of the induced current obtained on the induction electrode during the step process. The peak of the time-domain waveform of the electrostatic signal coincides with the moments when the foot separates from the initial contact with the ground. The moments of foot separation from and initial contact with the ground can be obtained by extracting the corresponding peak information.

### 2.4. Subjects

The current study was approved by the institutional academic board. A total of 10 testers participated in the gait measurement (seven are male and three are female). They have an average weight of 64.8 kg (weight range: 45~77 kg), and an average height of 1.7 m (height range: 1.58~1.79 m). Exclusion criteria included neurological or lower extremities conditions, respiratory or cardiovascular problems, insanity or mental disorder, and pregnancy. All subjects were informed of the purpose as well as the methods and instructions to complete the experiment measuring temporal gait parameters and all subjects signed informed consent paperwork.

### 2.5. Experimental Conditions

In the experiment, the insoles carrying the foot pressure system were placed under the right foot of the testers. The battery and conditioning circuit were fixed to the middle of the calf using the elastic band and the testers wore their own shoes to ensure the most natural movement. At the same time, the testers needed to keep the coronal plane parallel to the electrostatic induction electrode (shown in Figure 3) during walking. Testers were required to carry out the stepping movement at a slow, normal, and fast pace. An electronic metronome was used to assist testers in completing this process. In the course of the experiment, the testers were required to implement each type of pace continuously for at least 20 s in order to collect enough gait data. Since the human body needs to have an adjustment period before entering a steady step, 2 s at the beginning and 2 s at the end of each data point were removed to ensure data validity. Lastly, 300 valid gait cycle data were obtained from the EFS installation and the foot pressure system. At the same time, to rule out the effect of temperature and humidity on the electrostatic sensing method, the whole experiment was carried out at a laboratory temperature of 25 °C and relative humidity (RH) of 55%.

### 2.6. Analysis

The concurrent validity of temporal gait parameters obtained by the EFS method and by the foot pressure system was assessed using Pearson correlation coefficients. In addition, intraclass correlation coefficients (ICC) (2,1) (two-way random effect, single measure model) were used to assess the test-retest reliability of the temporal gait parameters obtained by the electrostatic sensing method between day 1 and day 8. All analyses were conducted with *p* < 0.05 as the significance level and performed using SPSS Version20.0 (IBM Corporation, Armonk, NY, USA). For the Pearson coefficient *r*, an excellent relationship was considered if *r* was greater than 0.90, a good relationship if *r* was between 0.8 and 0.89, a fair relationship if *r* was between 0.7 and 0.79, and a poor relationship if *r* was below 0.70 [35]. Regarding the ICC, it was considered excellent if the ICC was greater than 0.90, good if the ICC was between 0.75 and 0.90, moderate if the ICC was between 0.50 and 0.75, and poor if the ICC was below 0.50. The data processing and the algorithms were implemented in Matlab (R2016b, MathWorks Inc., Natick, MA, USA).

## 3. Results

According to the principle in Section 2.3, we utilized Matlab to process the data from the foot pressure system and the EFS method. We extracted the initial contact (IC) and separate events (SE) according to the proposed algorithm. The measurement error of the EFS method was verified with the date measured by the foot pressure system. Data analysis showed that, in the judgment of initial contact (IC) with the ground, the measurement error of the EFS method is in the range of ±40 ms (mean value: −1.5 ± 15 ms). In the judgment of separate events (SE) from the ground, the measurement error of the EFS method is also within ±40 ms (mean value: −1.3 ± 15 ms).

**Figure 6:**
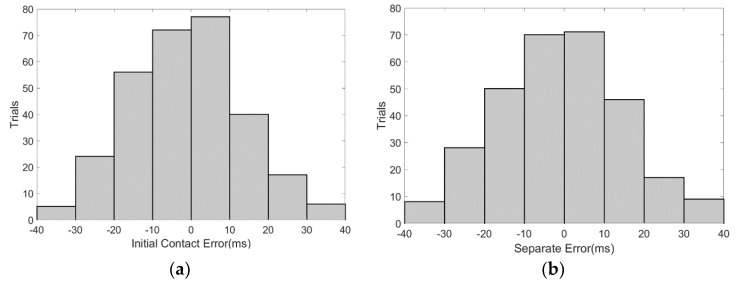
Measurement error of the moments of foot contact with and separation from the ground by the electrostatic field sensing (EFS) method. (**a**) Initial contact (IC) error statistical histogram; (**b**) separate events (SE) error statistical histogram.

We counted the difference between moments obtained by the EFS method and the foot pressure system in 300 cycles of 10 testers, the statistical histogram of which is shown in Figure 6a,b.

Once gait events are identified, gait cycle (*T_g_*), stance phase duration (*T_s_*), swing phase duration (*T_w_*), and gait cadence (*C*) can be calculated, as shown in Equations (5)–(8).

(5) $$T_{g}=IC(m+1)-IC(m)$$

(6) $$T_{s}=SE(n)-IC(m)$$

(7) $$T_{w}=IC(m+1)-SE(n)=T_{g}-T_{s}$$

(8) $$C=\frac{60}{T_{g}}\times 2\;(\mathrm{Steps}/\mathrm{Min})$$

The ratio of *T_s_* over *T_g_* (*R_S_*) and the ratio of *T_w_* over *T_g_* (*R_W_*) are then computed. These ratios should represent approximately 60% of the stance phase duration (*R_S_* ≈ 60%) and 40% of the swing phase duration (*R_W_* ≈ 40%) if a person has a healthy gait. 

**Figure 7:**
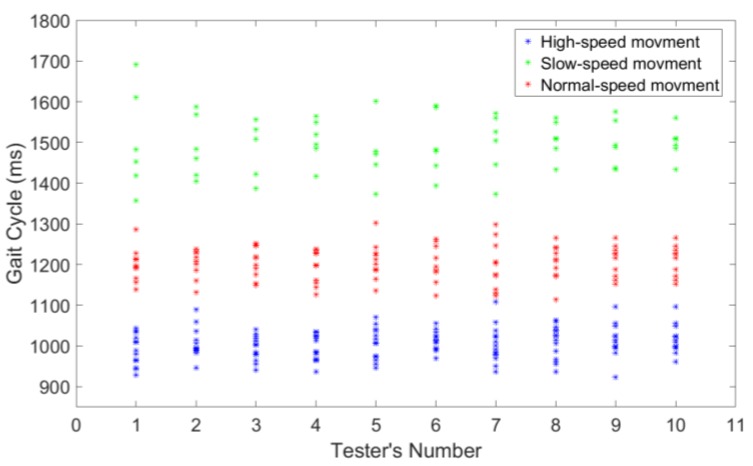
Gait cycle of the 10 testers.

Therefore, the gait cycle (*T_g_*) of 10 testers at three different motion speeds are calculated and counted. The gait cycle (*T_g_*) measured by the foot pressure system during high-speed, normal-speed, and slow-speed movement are 990 ± 120 ms (range: 870~1153 ms), 1198 ± 110 ms (range 1088~1398 ms), and 1498 ± 75 ms (range: 1358~1697 ms), respectively. The gait cycle derived from the 10 testers is illustrated separately in Figure 7.

We measured the error of the EFS method in the gait cycle (*T_g_*) measurement with the foot pressure system. The error and the error percentage during high-speed, normal-speed, and low-speed movement are −0.2 ± 18 ms (range of ±45 ms) and 0.05 ± 2% (range of ±4%), 0.3 ± 18 ms (range ±39 ms) and 0.04 ± 2% (range of ±3%), and 1 ± 13 ms (range of ±30 ms) and 0.07 ± 1% (range of ±2%), respectively. This showed an average accuracy rate of 97%.

**Figure 8:**
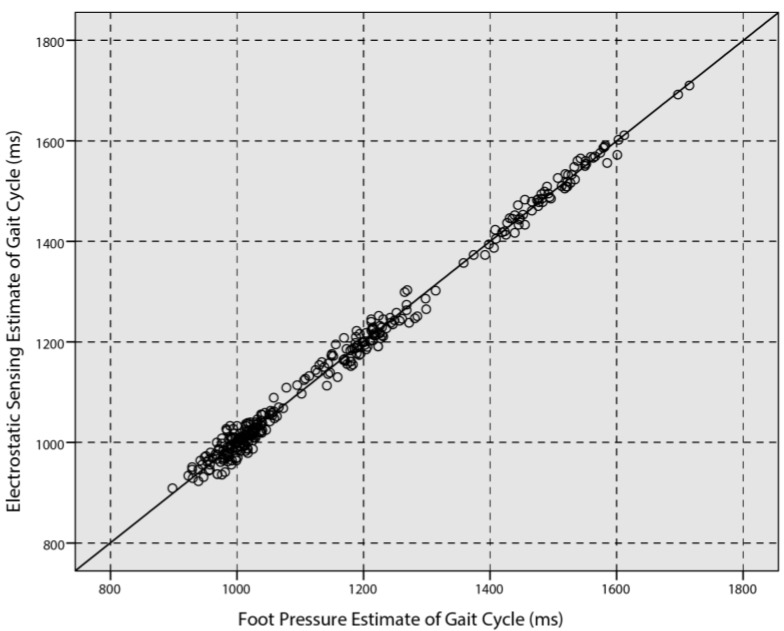
Gait cycle (*T_g_*) obtained by the EFS method and the foot pressure system.

The EFS-derived estimate of the gait cycle (*T_g_*) agreed with the foot pressure system over the entire range of walking rates (see Figure 8). The two estimates of the gait cycle (*T_g_*) were strongly correlated (Pearson’s correlation coefficient *r* = 0.99). This confirmed the high reliability of the electrostatic sensing-based estimate of the gait cycle.

Table 1 shows the statistical results of the electrostatic field sensing (EFS) method and the foot pressure system of the other three temporal gait parameters. The concurrent validity of the EFS method and the foot pressure system showed that the two methods obtained excellent correlation in these three gait parameters.

Apart from these results, further validation showed that the average *R_S_* for the EFS method is 60.49 ± 2.45%. This value is in accordance with the norm gait parameters in which the stance phase lasts approximately 60% of the gait cycle for healthy individuals. Based on these results, it can be deduced that the proposed method can accurately determine temporal gait parameters.

**Figure 9:**
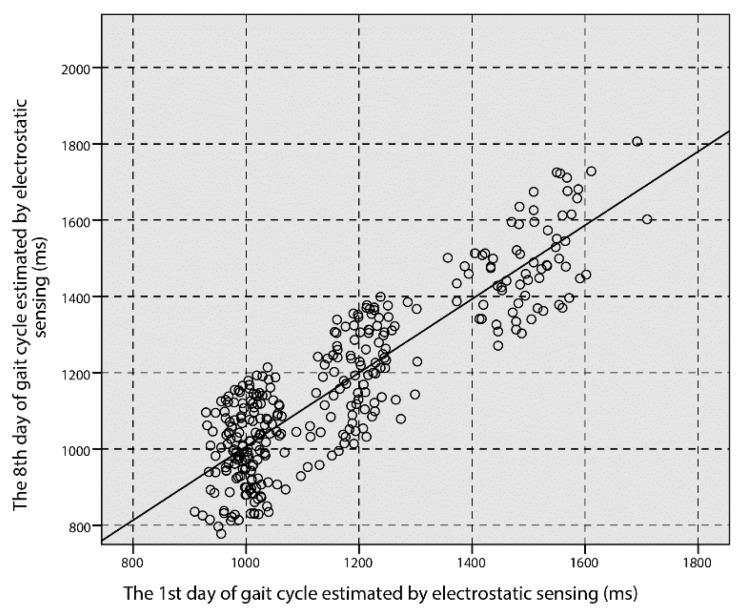
EFS method estimation of the gait cycle for day 1 and day 8.

The test-retest reliability of the EFS method on the first and eighth days shows that the ICC is 0.87 (*p* < 0.001). The scatter plot of the gait cycle measured using the EFS method for the first day and the eighth day is shown in Figure 9. The ICC values of the remaining gait parameters are illustrated in Table 2.

## 4. Discussions

Most existing gait event measurement methods rely on mechanical or kinetic parameters derived from an optical motion capture system and force plate. However, these devices rely on professional analyses to obtain gait parameters and can only operate in a confined space. These methods also require testers to control walking speeds and step length to ensure that the subject’s foot always comes in contact with the force plate. Data obtained from these methods cannot be fully reflected in a natural human gait. On the other hand, wearable sensors can meet gait measurement needs under non-laboratory conditions. However, they all require placing sensors on the subject’s body, which may be uncomfortable or obtrusive. Therefore, a low-cost, non-wearable EFS method can be a promising alternative for monitoring and determining gait temporal parameters of individuals outside the laboratory environment.

Previous research [25,33] employed the EFS method to detect sports motion signals and presented a method to recognize the moments of foot contact with a separation from the ground. However, the study of extracting temporal gait parameters forming the FES signal has not been explored. In addition, the credibility and test-retest repeatability of this method has not been verified. An algorithm for extracting temporal gait parameters from gait EFS signals was presented in this paper. The measuring error of the EFS method was calibrated with the foot pressure system. The study results showed that temporal gait parameters could be identified through EFS signals in the stepping gait and two important gait cycle parameters (initial contact and separate event) could be detected with high accuracy. Based on these detected results, six types of temporal gait parameters were calculated. These calculated parameters fit well with the results measured by the foot pressure system.

Our proposed EFS method manifested excellent correlation coefficients to the foot pressure system’s results (*r* = 0.99, *p* < 0.05) and test-retest reliability (ICC = 0.87, *p* < 0.001) across an eight-day test-retest interval. These results indicate that the suggested EFS method may be a reliable measure of temporal gait parameters.

Kluge et al. employed an inertial measurement system to evaluate temporal gait parameters [36]. Two Shimmer3 sensors, which contained a three-axis gyroscope and a three-axis accelerometer worn only on the feet, obtained the temporal gait parameters. A state-of-the-art algorithm was used to extract the temporal gait parameters. The concurrent validity of the proposed system was compared with an external camera-based system. Even though their method obtained satisfactory precision in measuring stride time, the error was 5.4% when measuring the stance time. Contrary to the results of Kluge et al., our method has an average of 3% error in measuring stance time, which shows better measurement accuracy. Moreover, the method by Kluge et al. is a contact measuring system, which may reduce the comfort of the tester.

Cheng et al. used a wearable microphone-sensor-based system to collect footstep sound signals during walking [37]. Based on this system, a gait analysis algorithm was proposed for estimating the temporal parameters of gait. Although the purposed method used a non-contact acoustic signal detection method, the ambient sound noise and sound delay affected the measurement accuracy. The results showed that only 94.52% of the average accuracy could be obtained when measuring initial contact. In addition, although a non-contact microphone sensor was used, the tester still was required to wear the measurement system when testing. Our method uses the variable electrostatic field information to obtain the gait signal, which has a higher signal-to-noise ratio with a smaller time delay.

Yong developed a low-cost wearable wireless ultrasonic sensor system for estimating three-dimensional displacement to extract temporal gait parameters [38]. The lightweight and miniaturized sensor design could make the patient more unconstrained. Their system had only 3% measurement error when measuring the stride duration, which basically is the same accuracy as that achieved with our electrostatic sensing method. However, this ultrasonic-based gait parameter measurement method relied on a complex signal processing algorithm and lacked a visual representation of the gait signal. In our study, the induced current signal is strongly correlated with the gait and the algorithm for extracting the temporal gait parameters is simple.

The results showed that the EFS method can accurately measure the initial and separate events of walking as well as effectively obtain temporal gait parameters. The limitation of this study is that we only used a single sensor to estimate gait parameters. A single sensor has a certain effective measurement range and cannot capture gait parameters beyond the effective measurement range. The present algorithm can only analyze a single tester’s gait data and cannot analyze the gait data of multiple testers in the effective measurement range simultaneously. Moreover, we lack gait data of real neurological patients. However, if the patient is able to completely lift his feet off the ground in the gait cycle, our method could capture gait events and then calculate the gait parameters. The differences between gait parameters could be used to diagnose related neurological diseases. Despite this limitation, our low-cost, wireless, non-wearable method and estimation algorithm were proven to be effective and valuable for calculating temporal gait parameters.

With the rise of telemedicine, medical measurement systems will lead to major change. Clinicians and researchers are working toward long-distance measurement methods to monitor human gait parameters and provide remote diagnosis and guidance. This method could assist health professionals to monitor patients’ behavior and prescribe corrective action when performing their activities of daily living. Although the method presented in this article is capable of detecting several gait parameters, we will also investigate the potential of expanding this method to extract more gait information or automatically start the measurement to complete the data capture and gait analysis when the patients enter the effective sensing area.

## 5. Conclusions

In this study, we presented a temporal gait parameters estimation method based on EFS, which uses human walking-generated electrostatic field information. For the first time, the temporal gait parameters were obtained based on electrostatic field sensing. By comparing the results with those obtained by a foot pressure system, the validity and test-retest reliability were verified. Due to the advantages of non-wearable, low-cost, long-term uninterrupted measurement, the proposed method could provide an affordable and accurate tool for conducting human gait parameters measurement in clinical laboratories and patients’ home environments.

## Figures and Tables

**Table 1:** The statistical results of the EFS method and the foot pressure system with the Pearson coefficient value of three gait parameters (*p* < 0.05).

Gait Parameters	EFS Result	Foot Pressure Result	Pearson Coefficient *r*
Stance phase duration (*T_s_*)	741.83 ± 117.97	775.03 ± 125.68	0.98
Swing phase duration (*T_w_*)	431.32 ± 94.12	396.58 ± 94.10	0.99
Gait cadence (*C*)	102.53 ± 15.34	102.66 ± 15.42	0.99

**Table 2:** The statistical results of the EFS method results on the first and eighth days as well as the intraclass correlation coefficient (ICC) values of gait parameters.

Gait Parameters	Day 1	Day 8	ICC	*p*
Stance phase duration (*T_s_*)	741.83 ± 117.97	805.56 ± 120.64	0.86	<0.001
Swing phase duration (*T_w_*)	431.32 ± 94.12	458.79 ± 102.35	0.87	<0.001
Gait cadence (*C*)	102.53 ± 15.34	95.23 ± 16.49	0.85	<0.001
